# Structural insights into the cross-neutralization of SARS-CoV and SARS-CoV-2 by the human monoclonal antibody 47D11

**DOI:** 10.1126/sciadv.abf5632

**Published:** 2021-06-02

**Authors:** Juliette Fedry, Daniel L. Hurdiss, Chunyan Wang, Wentao Li, Gonzalo Obal, Ieva Drulyte, Wenjuan Du, Stuart C. Howes, Frank J.M. van Kuppeveld, Friedrich Förster, Berend-Jan Bosch

**Affiliations:** 1 Cryo-Electron Microscopy, Bijvoet Center for Biomolecular Research, Department of Chemistry, Faculty of Science, Utrecht University, Padualaan 8, 3584 CH Utrecht, Netherlands.; 2 Virology Section, Infectious Diseases and Immunology Division, Department of Biomolecular Health Sciences, Faculty of Veterinary Medicine, Utrecht University, 3584 CL Utrecht, Netherlands.; 3 Crystal and Structural Chemistry, Bijvoet Center for Biomolecular Research, Department of Chemistry, Faculty of Science, Utrecht University, Padualaan 8, 3584 CH Utrecht, Netherlands.; 4 Materials and Structural Analysis, Thermo Fisher Scientific, 5651 GG Eindhoven, Netherlands.

## Abstract

The emergence of SARS-CoV-2 antibody escape mutations highlights the urgent need for broadly neutralizing therapeutics. We previously identified a human monoclonal antibody, 47D11, capable of cross-neutralizing SARS-CoV-2 and SARS-CoV and protecting against the associated respiratory disease in an animal model. Here, we report cryo-EM structures of both trimeric spike ectodomains in complex with the 47D11 Fab. 47D11 binds to the closed receptor-binding domain, distal to the ACE2 binding site. The CDRL3 stabilizes the N343 glycan in an upright conformation, exposing a mutationally constrained hydrophobic pocket, into which the CDRH3 loop inserts two aromatic residues. 47D11 stabilizes a partially open conformation of the SARS-CoV-2 spike, suggesting that it could be used effectively in combination with other antibodies targeting the exposed receptor-binding motif. Together, these results reveal a cross-protective epitope on the SARS-CoV-2 spike and provide a structural roadmap for the development of 47D11 as a prophylactic or postexposure therapy for COVID-19.

## Teaser

Cryo-EM reveals the molecular basis of SARS-CoV-2 cross-neutralization by the monoclonal antibody 47D11.

## INTRODUCTION

The severe acute respiratory syndrome coronavirus 2 (SARS-CoV-2) emerged from a zoonotic event in China, late 2019 (*1*). As of 4 February 2021, the resulting coronavirus-induced disease 19 (COVID-19) pandemic has been responsible for more than 100 million infections and more than 2 million deaths (https://covid19.who.int/). SARS-CoV-2 and SARS-CoV, another highly lethal respiratory pathogen that emerged in 2002/2003 (*2*), belong to the subgenus *Sarbecovirus* (genus *Betacoronavirus*, family Coronaviridae) (*3*). There is an urgent clinical need for potent antiviral therapies to halt the spread of SARS-CoV-2 and to preempt future outbreaks caused by SARS-like viruses. Antibodies are a promising class of drugs for combatting infectious diseases and have shown therapeutic efficacy for a number of viruses (*4*, *5*), including in the treatment of SARS and COVID-19 (*6*, *7*). Such antibodies function by targeting vulnerable sites on viral surface proteins.

The coronavirus trimeric spike (S) glycoprotein, located on the viral envelope, is the key mediator of viral entry into host cells. The spike protein is made of two subunits: S1 is involved in receptor binding and S2 in membrane fusion. The S1 subunit itself is further subdivided into an N-terminal domain (NTD; or S1_A_) and a receptor binding domain (RBD; or S1_B_) (*8*, *9*). The spike proteins of SARS-CoV-2 (SARS2-S; 1273 residues, strain Wuhan-Hu-1) and SARS-CoV (SARS-S; 1255 residues, strain Urbani) exhibit 77.5% identity in their amino acid sequence and are structurally conserved (*10*–*13*). The spike trimer exists in equilibrium between a closed conformation, where all three RBDs lie flat in a “down” conformation, and a partially open conformation, where one RBD adopts an “up” conformation and is exposed for receptor engagement (*10*–*13*). Both viruses use the human angiotensin converting enzyme 2 (ACE2) protein as a host receptor, with binding mediated through interactions with the receptor-binding motif (RBM) located on the RBD, and the N-terminal helix of ACE2 (*14*). The spike-mediated fusion of viral and cellular membranes is tightly regulated and triggered by a cascade of preceding events. The first step involves the attachment of SARS-CoV-2 to the target cell surface via the interaction between the spike and ACE2 (*14*, *15*). In the second step, the spike protein needs to be primed for membrane fusion by host proteases (e.g., cellular transmembrane serine protease 2), which cleave the spike at multiple sites (*16*), enabling shedding of S1. Last, the free S2 catalyzes the fusion of the viral and the host membranes (*17*, *18*), causing the release of the viral genome into the host cell cytoplasm.

The S glycoprotein is the primary target for neutralizing antibodies, making it the main target for vaccine development (*19*). A number of SARS-CoV-2–neutralizing antibodies have now been described (*20*–*34*). The most commonly identified antibodies neutralize coronaviruses by binding to the RBM in S1, blocking receptor interactions and/or promoting a premature S1 shedding and conformational change of spike to the postfusion state. However, a number of recently identified SARS-CoV-2 variants [B.1.1.7 (*35*), B.1.351 (*36*), B.1.1.28.1 (P.1) (*37*), and B.1.1.28.2 (P.2) (*38*, *39*)] harbor mutations in the RBM (K417N/T, E484K, and N501Y), which could facilitate viral escape from monoclonal antibodies (mAbs) binding to this region (*40*–*42*), as well as some polyclonal sera dominated by this class of antibodies (*43*). Fewer antibodies have been reported to bind epitopes that are distal to the ACE2 binding site. Such antibodies target the RBD core (*28*, *44*–*47*) or the NTD (*48*).

Cross-neutralizing antibodies are highly valuable in the development of antiviral therapeutics as they confer a broader protection and mitigate the risk of immune escape. However, conserved SARS-CoV and SARS-CoV-2 cross-protective epitopes appear to be rarely targeted by neutralizing antibodies (*44*–*47*, *49*), and only a handful of cross-neutralizing antibodies have been structurally characterized (*44*–*47*). Combined structural and functional studies are thus required to delineate epitopes eliciting cross-neutralizing antibodies and guide vaccine and antiviral development applicable to a wide range of future SARS-CoV-2 variants in the treatment of COVID-19 (*50*).

We recently reported the potent human mAb, 47D11, capable of cross-neutralizing SARS-CoV and SARS-CoV-2 at 1.3 and 3.8 nM, respectively, without competing with ACE2 binding (*51*). Subsequent preclinical studies revealed 47D11 prophylactic potential to prevent SARS-CoV-2–induced pneumonia in a hamster model (*52*). Here, we used structural and functional studies to decipher the molecular basis for 47D11-mediated cross-neutralization.

## RESULTS AND DISCUSSION

### 47D11 specifically recognizes the down conformation of the RBD

**Fig. 1:**
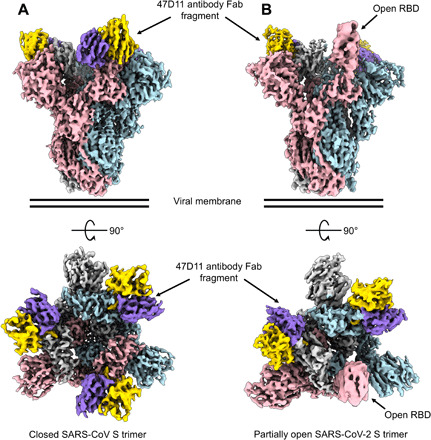
47D11 has differing conformational selectivity for the SARS-CoV and SARS-CoV-2 spike. (**A**) Surface rendering of the fully closed SARS spike bound to three 47D11 antibody Fab fragments, shown as two orthogonal views. (**B**) Surface rendering of the partially open SARS2 spike in complex with two 47D11 antibody Fab fragments, shown as two orthogonal views. The spike protomers are colored pink, blue, and gray, and the 47D11 HC and LC are colored yellow and purple, respectively. For clarity, only the Fab variable region is shown.

To understand how 47D11 binds to the SARS-CoV and SARS-CoV-2 spike proteins, we used cryo–electron microscopy (cryo-EM) single-particle analysis to determine structures of prefusion stabilized ectodomain trimers in complex with the 47D11 Fab fragment. The resulting cryo-EM maps have global resolutions of 3.8- and 4.0-Å for SARS-S and SARS2-S, respectively (fig. S1, A to F). For previously reported apo S trimers, both the open and closed conformations are observed, with the latter being predominant [56% for SARS-S (*11*) and 67% for SARS2-S (*12*)]. Upon incubation with 47D11, only the closed conformation of the SARS-CoV spike was observed, with stoichiometric binding of 47D11 to each RBD (Fig. 1A). For SARS-CoV-2, only the partially open conformation of the spike was observed, with one Fab bound to each of the two RBDs in down conformation and the remaining RBD in up conformation unoccupied and, in principle, accessible to ACE2 binding (Fig. 1B). The substoichiometric binding observed for SARS2-S may partially explain our previous observations that 47D11 binds to the SARS-S with higher affinity than SARS2-S [equilibrium dissociation constant (*K*_D_) of 0.745 and 10.8 nM, respectively] (*51*).

**Fig. 2:**
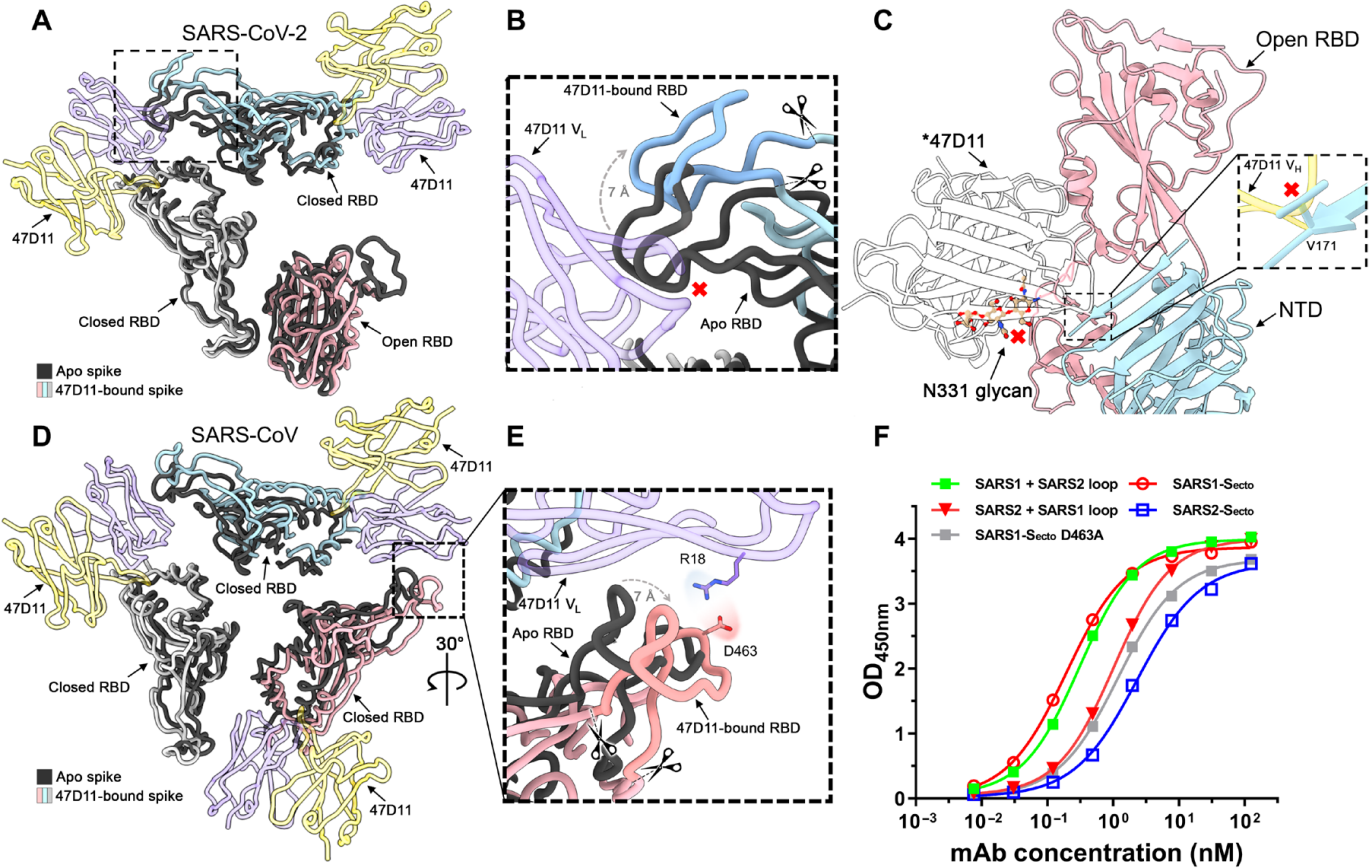
47D11 binds specifically to the RBD in down conformation and prevents their full compaction. (**A**) Top view of the 47D11-bound SARS2 spike, shown as a ribbon diagram. The 47D11-bound spike protomers are colored pink, blue, and gray, and the 47D11 HC and LC are shown semitransparently and colored yellow and purple, respectively. Glycans and the NTD are omitted for clarity, and only the Fab variable region is shown. The superposed structure of the partially open apo SARS2 spike (PDB ID: 6ZGG) is colored black. (**B**) Zoomed-in view of the boxed region in (A). The region encompassing residues 470 to 490, used for the loop swap experiments, is indicated with scissors. (**C**) Zoomed-in view of the SARS2 up RBD and adjacent NTD, shown in cartoon representation. The overlaid 47D11 Fab is shown as a silhouette, and the N331 glycan is shown in ball-and-stick representation and colored tan. The inset shows a zoomed-in view of the clash between the NTD residue V171 and the 47D11 HC. (**D**) Top view of the 47D11-bound SARS spike colored as shown in (A). The superposed structure of the closed apo SARS spike (PDB ID: 5XLR) is colored black. (**E**) Zoomed-in view of the boxed region in (D), showing a putative salt bridge between the 47D11 variable LC and the RBD loop. The region encompassing residues 457 to 477, used for the loop swap experiments, is indicated with scissors. (**F**) ELISA binding curves of 47D11 binding to wild-type, loop-swapped, and D463A spike ectodomains. V_H_, variable region of immunoglobulin heavy chain; V_L_, variable region of immunoglobulin light chain; OD_450nm_, optical density at 450 nm.

To understand why 47D11 favors different spike conformations for SARS-CoV and SARS-CoV-2, we first superposed the Fab-bound structures on their apo counterparts. Compared to the apo partially open SARS2-S structure, the RBDs are less compact when 47D11 is bound (Fig. 2A). The apo down conformation of the RBD would preclude binding of 47D11 to the adjacent RBD through steric hindrance. To accommodate the bound Fab on the latter, the first RBD shifts outward by ~7 Å (Fig. 2B). Unlike mAbs S309 and H014 (*44*, *47*), two other RBD core–targeting cross-neutralizing antibodies, there was no indication from our cryo-EM data that 47D11 can bind to the up conformation of the SARS2-S RBD. In line with this observation, superimposition of the closed and open SARS2-S RBDs revealed that 47D11 would clash with the adjacent NTD and the N331 glycan in the latter conformation (Fig. 2C).

Similar to SARS2-S, the RBDs of the 47D11-bound SARS-S are also less compact than the reported apo fully closed structure (Fig. 2D). In contrast to SARS2-S, there is a potential stabilizing salt bridge between SARS-S D463, located on the receptor binding ridge, and R18 on the 47D11 light chain (Fig. 2E). This ridge exhibits the most prominent structural differences between SARS2-S and SARS-S (*14*). This epitope distal loop, located within the ACE2 binding region, contains an essential disulfide bridge in both viruses but is more compact in SARS-S. To test whether the epitope distal ridge affects binding of 47D11 to the SARS-S and SARS2-S, we swapped loop residues 470 to 490 (SARS2-S numbering) and produced chimeric ectodomains. We also introduced a D463A mutation in SARS-S to disrupt the observed salt bridge (Fig. 2E). In support of our hypothesis, the SARS2-S containing the SARS-S loop exhibited increased binding to 47D11, whereas the SARS-S D463A mutant displayed decreased binding to 47D11 and loss of ACE2 binding (fig. S4, A and B), consistent with previous reports (*53*). However, we did not observe an equivalent loss of binding for the chimeric SARS-S, suggesting that other differences in protein sequence or quaternary structure may be involved (Fig. 2F). Together, our data show that 47D11 binding to the RBD has differing outcomes for SARS-S and SARS2-S, trapping them in the fully closed and the partially open conformation, respectively (fig. S2).

These results rationalize our previous observation that the 47D11-bound SARS2-S can still bind soluble ACE2 in a cell staining assay (*51*), given that it has one open RBD accessible to the receptor. It is unclear, however, how the 47D11-bound SARS-S, stabilized with the three RBDs in the down conformation, can also bind ACE2 (*42*). The 47D11-bound RBDs may be able to adopt a semi-open conformation, too transient to be visualized by cryo-EM, which could accommodate both 47D11 and ACE2 binding (*54*). Alternatively, it is possible that 47D11 binding results in destabilization of the spike trimer leading to the separation of the protomers and exposure of the ACE2 binding site, as reported for CR3022 (*55*). Similarly to S309 (*35*), the absence of competition between 47D11 and ACE2 complicates the determination of the exact neutralization mechanism. First, as viral membrane fusion is a tightly controlled process, it is possible that the perturbation of the spike conformational flexibility induced by 47D11 binding hinders the correct and timely S1 shedding and subsequent conformational changes required for fusion. It is also possible that immunoglobulin G (IgG)–specific bivalent mechanisms such as spike cross-linking, steric hindrance, or virus aggregation are involved. Ultimately, further studies are required to fully decipher the neutralization mechanisms of 47D11.

### 47D11 targets a conserved hydrophobic pocket in the RBD

**Fig. 3:**
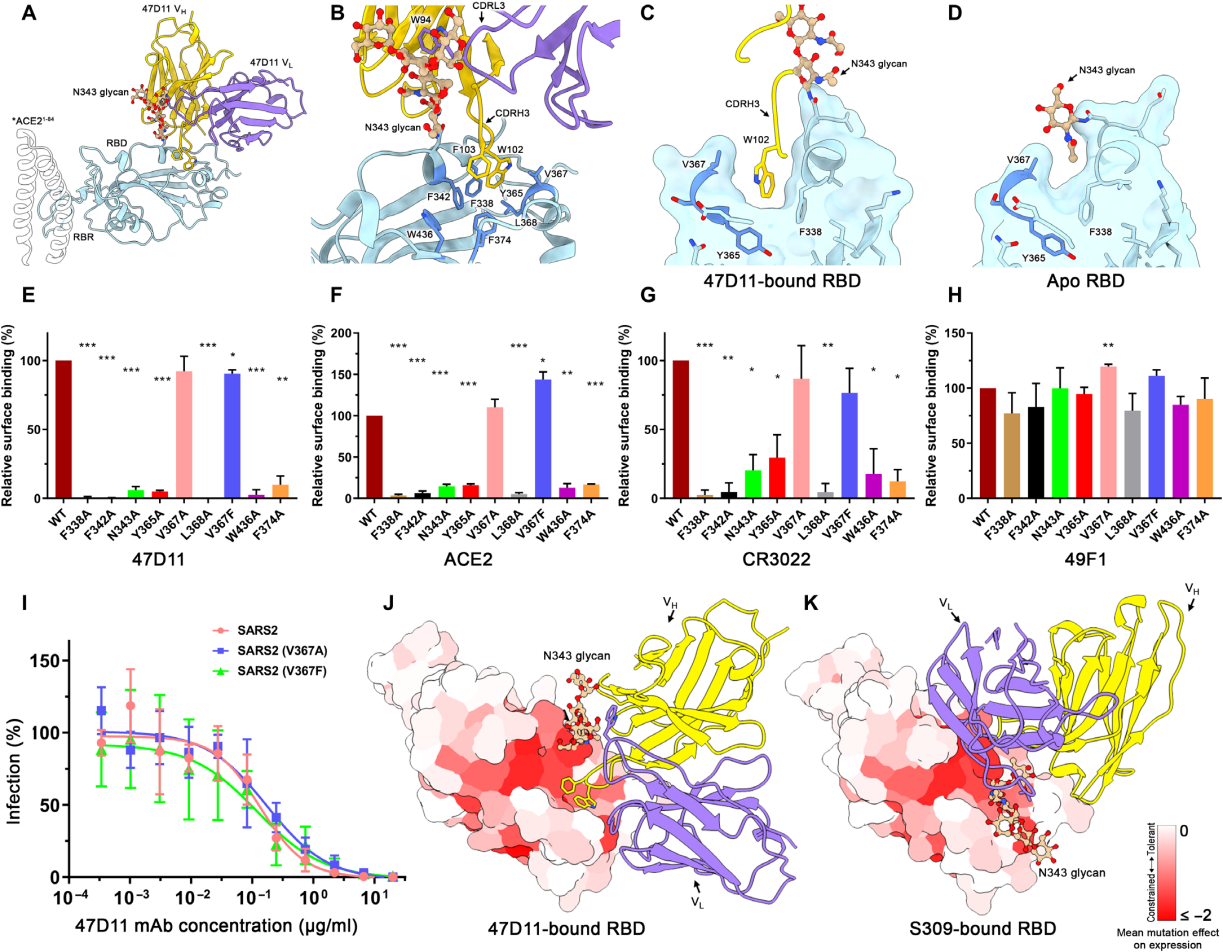
The 47D11 epitope comprises a mutationally constrained hydrophobic pocket that is normally shielded by glycan N343. (**A**) Ribbon diagram of the SARS2-S RBD in complex with the 47D11 antibody Fab fragment. For comparison, residues 1 to 84 of the RBD-bound ACE2 (PDB ID: 6M0J) are shown as a silhouette. (**B**) Close-up view of the 47D11 epitope with the hydrophobic pocket residues shown as sticks and colored dark blue. The N343 glycan is shown in ball-and-stick representation and colored tan. For clarity, only the core pentasaccharide is shown. (**C**) Slice through the surface rendered 47D11-bound SARS2-S RBD. The helix encompassing residues 365 to 370 is shown in darker blue. (**D**) Equivalent view as shown in (C) for the apo RBD (PDB ID: 6VYB). (**E**) Relative binding of 47D11, (**F**) ACE2, and (**G**) CR3022 to cell surface–expressed SARS2-S, determined by fluorescence-activated cell sorting. (**H**) Relative binding of an anti-FLAG antibody to permeabilized cells expressing the full-length SARS2 spike epitope mutants, determined by fluorescence-activated cell sorting. The data were analyzed by the unpaired, two-tailed Student’s *t* test using GraphPad Prism 7.0. *P* < 0.05 was considered significant (*P < 0.05, **P < 0.01, and ***P < 0.0001). (**I**) Antibody-mediated neutralization of infection of luciferase-encoding VSV particles pseudotyped with wild-type, V367A, or V367F SARS2-S. (**J**) Surface representation of the 47D11-bound SARS2-S RBD colored according to mean mutation effect on expression (red indicates more constrained) (*58*). The Fab is shown as a ribbon diagram. (**K**) As shown in (E) for the S309-bound SARS2 RBD.

The 47D11 epitope is distinct from the ACE2 binding site (Fig. 3A), rationalizing its ability to cross-neutralize SARS-CoV and SARS-CoV-2 independently of receptor-binding inhibition (*51*). The protein/glycan epitope is located on the core domain of the SARS-S and SARS2-S RBD. As expected, the mode of binding is highly similar for SARS-S and SARS2-S (fig. S3, A to C), with the aligned 47D11:RBD complexes deviating by a root mean square deviation (RMSD) value of 1.4 Å per 201 Cα atoms.

The paratope is composed of CDRL3 and CDRH3 loops, which form a primarily hydrophobic interaction burying an RBD surface area of ~830 and ~800 Å^2^ for SARS-S and SARS2-S, respectively. The side chain of 47D11 CDRL3 tryptophan W94 stacks against the N330/N343 (SARS/SARS2) glycan tree, contributing to its stabilization in an upright conformation (Fig. 3B). The structure revealed a hydrophobic pocket into which the CDRH3 loop projects, allowing Fab residues W102 and F103 to interact with RBD core residues F338, F342, Y365, V367, L368, F374, and W436 (F325, F329, Y352, V354, L355, F361, and W423 in SARS-S) (Fig. 3B and fig. S3B). This pocket is shielded by the N343 glycan in previously reported apo SARS2-S structures (Fig. 3, C and D) (*12*, *13*).

To accommodate the CDRH3 loop residues, the helix encompassing residues 365 to 370 is displaced outward by 2 Å, creating 55 Å^3^ of solvent-accessible volume, which is not present in the apo RBD (Fig. 3, C and D, and fig. S3, E and F). Notably, the region directly below this hydrophobic pocket was recently shown to bind to linoleic acid, which stabilizes the closed conformation of the spike by spanning two adjacent RBDs (*56*). In the 47D11-bound RBD, the position of the helix encompassing residues 365 to 370 would preclude binding of linoleic acid (fig. S3E). Consistent with this arrangement, no density corresponding to linoleic acid could be detected in any of our reconstructions.

To verify the 47D11 epitope, we introduced single alanine mutations into full-length SARS2-S and expressed these at the surface of human embryonic kidney (HEK) 293T cells. In addition, we generated a spike mutant with the naturally occurring V367F minority variant (*57*). Total cellular expression of mutants was comparable to wild-type spike protein as demonstrated by an antibody targeting the C-terminal appended Flag-tag on the spike proteins (fig. S4C). Binding of 47D11 to cell surface–expressed wild-type and mutant spike proteins was assessed by flow cytometry. As controls, we used the soluble Fc-tagged ACE2, as well as the RBD core–targeting mAb CR3022 (*28*) and the mAb 49F1, which binds S1 outside the RBD (*51*). Similar binding levels of the 49F1 antibody in wild-type and mutant spike proteins confirmed the correct cell surface localization of the mutants (Fig. 3H). Mutations of V367 to a phenylalanine or an alanine had only a minor effect on 47D11 antibody binding (Fig. 3E), consistent with data showing that this polymorphism had no effect on neutralization of SARS2-S pseudotyped virus (Fig. 3I). Collectively, these data indicate that 47D11 would be effective against this SARS-CoV-2 variant. In contrast, all other amino acid substitutions in the hydrophobic core not only reduced 47D11 binding (Fig. 3E) but also prevented binding of ACE2 and of the core-targeting antibody CR3022, despite being distal to their respective interaction sites on the RBD (Fig. 3, F and G, and fig. S5, A and B). These results suggest that these mutations have an effect on the tertiary structure of the whole RBD including the distal ACE2 binding ridge.

In line with this explanation, a recent study reported deep mutational scanning of SARS2-S RBD residues, revealing how the mutation of each of the RBD residues affects the expression of folded protein and its affinity for ACE2 (*58*). When the mean mutation effect on expression was mapped on the 47D11-bound RBD, we observed that the hydrophobic pocket, targeted by 47D11, is highly mutationally constrained (Fig. 3J). Together, the mutational space in the 47D11 epitope region appears to be strongly limited by a concomitant loss of ACE2 binding, possibly lowering the risk of immune escape.

The 47D11 epitope matches a region of the RBD earlier described as relatively “immune silent” (*46*). It is distinct from other reported RBD core–targeting antibodies/nanobodies, such as CR3022, H014, and VHH-72 (fig. S5A) (*28*, *47*, *59*). Another SARS-CoV and SARS-CoV-2 cross-neutralizing antibody identified from the memory B cells of a patient with SARS-CoV, S309, targets a similar region to 47D11, but here, the orientation of the N343 glycan prohibits access to the hydrophobic pocket, similarly to apo structures (Fig. 3K and fig. S5A) (*44*). The quaternary epitopes of the SARS-CoV-2–neutralizing antibodies C144 (*60*) and S2M11 (*61*), isolated from COVID-19 recovering patients, also include the conserved hydrophobic pocket targeted by 47D11 (fig. S5B). However, the C144 and S2M11 epitopes extend to the RBM of the adjacent RBD, which is not conserved between SARS-CoV and SARS-CoV-2. Our data show that the 47D11 epitope, being restricted to a single RBD core, accounts for its cross-neutralization ability, while C144 and S2M11 fail to neutralize SARS-CoV.

### 47D11 neutralizes emerging SARS-CoV-2 variants

**Fig. 4:**
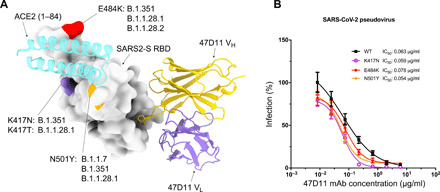
47D11 neutralizes SARS2-S pseudoviruses containing single mutations found in the RBM of recently emerged SARS-CoV-2 variants. (**A**) Surface representation of the 47D11-bound RBD with the location of the K417N/T, E484K, and N501Y mutations from SARS-CoV-2 variants colored purple, red, and orange, respectively, and annotated (*35*–*39*). The 47D11 Fab variable chains are shown as a ribbon diagram, and the HC and LC are colored yellow and purple, respectively. Residues 1 to 84 of the RBD-bound ACE2 (PDB ID: 6M0J) are shown for comparison and colored cyan. (**B**) 47D11-mediated neutralization of infection of luciferase-encoding VSV particles pseudotyped with SARS-CoV-2 S containing single RBM mutations. The average ± SD from two independent experiments with technical triplicates is shown.

Recently emerged SARS-CoV-2 variants carry RBD mutations, which localize to the ACE2-binding motif, namely K417N, E484K, and N501Y (Fig. 4A). While these mutations are distal to the 47D11 binding site, our data reveal a cross-talk between the RBM and the core region targeted by 47D11, as mutations in the 47D11 epitope resulted in loss of ACE2 binding (Fig. 3, F and G). Reciprocally, we analyzed the effect of emerging mutations in the RBM on 47D11’s neutralization ability by introducing the K417N, E484K, or N501Y mutation into SARS2-S pseudotyped vesicular stomatitis virus (VSV). Our data show that 47D11 neutralization efficiency is not affected by these single mutations in the RBM (Fig. 4B), making 47D11 a promising therapeutic candidate in the fight against the new, fast-spreading SARS-CoV-2 variants.

### 47D11 displays broader reactivity among at-risk bat coronaviruses

**Fig. 5:**
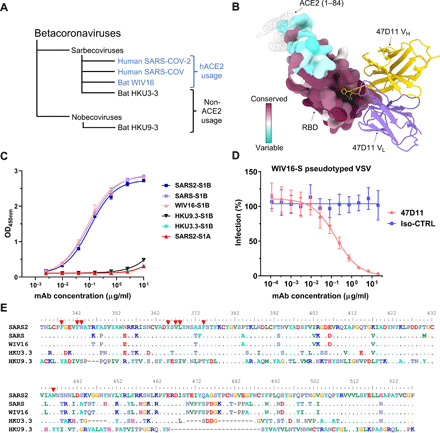
The 47D11 epitope is conserved in SARS-like viruses. (**A**) Phylogenetic tree of SARS-like viruses RBD used to assess 47D11 binding (*62*). (**B**) Surface representation of the 47D11-bound RBD colored according to sequence conservation across SARS-CoV, SARS-CoV-2, and 11 SARS-like viruses (fig. S5). The 47D11 Fab variable chains are shown as a ribbon diagram and colored gray. HC residues W102 and F103 are shown as sticks. For comparison, residues 1 to 84 of the RBD-bound ACE2 (PDB ID: 6M0J) are shown as a silhouette. (**C**) ELISA binding curves of 47D11 to the S1_B_ domain of SARS, SARS2, WIV16, HKU3-3, and HKU9-3. The average ± SD from two independent experiments with technical duplicates is shown. (**D**) 47D11-mediated neutralization of infection of luciferase-encoding VSV particles pseudotyped with WIV16-S. An anti–Strep-tag human mAb was used as an antibody isotype control. The average ± SD from two independent experiments is shown. (**E**) Aligned RBD sequences of SARS-CoV-2, SARS-CoV, WIV16, HKU3-3, and HKU9-3. Key residues in the 47D11 epitope are indicated by red arrowheads.

To assess whether 47D11 has broad reactivity, we analyzed 47D11 binding to the recombinantly expressed RBDs of three SARS-like bat betacoronaviruses: the sarbecoviruses WIV16 and HKU3-3 and the more distant nobecovirus HKU9-3 (Fig. 5A). Comparative sequence analysis revealed that the 47D11 epitope is highly conserved across circulating SARS-like sarbecoviruses (Fig. 5B and fig. S6). This is in contrast to the ACE2 binding region, which exhibits the greatest sequence variability. The results demonstrated that 47D11 binds to the WIV16 RBD with similar affinity to SARS-S and SARS2-S (Fig. 5C) and neutralizes WIV16-S pseudotyped VSV with a half-maximal inhibitory concentration (IC_50_) value of 0.165 μg/ml (Fig. 5D). In contrast, 47D11 does not bind the RBDs of HKU3-3 and HKU9-3 (Fig. 5C). HKU9-3 has the most distantly related RBD sequence to SARS-CoV-2, and the N343 glycosylation site as well as the hydrophobic residues of the 47D11 epitope are not conserved, explaining the lack of antibody binding (Fig. 5E). In both WIV16 and HKU3-3, the 47D11 epitope is conserved, but HKU3-3 displays four amino acid variations introducing charges in close proximity to 47D11: L335R, 339GE340 to DK, and N360D, which could preclude 47D11 binding. Notably, unlike WIV16, neither HKU3-3 nor HKU9-3 is able to bind human ACE2 (*62*–*64*). Sarbecoviruses (which include SARS-CoV, SARS-CoV-2, and numerous bat and a few pangolin viruses) are considered to be a high-risk group for potential emergence (*62*). Both sarbecoviruses that cause human disease, SARS-CoV and SARS-CoV-2, use the human ACE2 for cell entry, and hence, it is thought to be a trait of particular importance in the emergence pathway of sarbecoviruses (*62*). Our results provide a proof of principle that 47D11 could contribute to treatments for future outbreaks caused by ACE2-dependent SARS-like viruses.

In summary, our structural and functional studies demonstrate that 47D11 achieves cross-neutralization of the sarbecoviruses SARS-CoV-2 and SARS-CoV, by targeting a glycan-shielded, conserved pocket on the spike RBD. This cryptic site of vulnerability offers an attractive target for the design of cross-protective vaccines and targeted therapeutics. Genetic diversity of SARS-CoV-2 has recently increased, and the genetic/antigenic variation will rise further in time, as observed for the endemic human coronavirus HCoV-229E, which exhibits cumulative sequence variation in the RBD loops engaging its cellular receptor (*65*). The conserved nature of the glyco-epitope, evolutionary constrained by limited mutational space, may confer to the 47D11 antibody a sustainable applicability in neutralizing a wide range of future-emerging virus variants. Antibody combinations targeting nonoverlapping epitopes are currently of high interest as they may act synergistically, permitting a lower dosage and an increased barrier to immune escape (*61*). In this respect, and unlike C144 (*60*) and S2M11 (*61*), which lock the SARS2-S in its closed conformation, our structural data show that 47D11 stabilizes the partially open conformation of the SARS2-S. This may render the spike more susceptible to other mAbs, which target epitopes only exposed in the RBD up conformation, such as H014, CR3022, or antibodies targeting the ACE2 binding ridge, thus making 47D11 a prime candidate for combination treatment. Together, our results delineate a conserved vulnerability site on the SARS-CoV-2 spike and provide a fundamental insight to support the rational development of antibody-based interventions in the treatment of COVID-19.

## METHODS

### Expression and purification of coronavirus spike proteins

To express the prefusion spike ectodomain, gene encoding residues 1 to 1200 of SARS2-S (GenBank: QHD43416.1) with proline substitutions at residues 986 and 987, a “AAARS” substitution at the furin cleavage site (residues 682 to 685), and residues 1 to 1160 of SARS-S (GenBank: AAP13567.1) with proline substitutions at residues 956 and 957, a C-terminal T4 fibritin trimerization motif, and a StrepTag were synthesized and cloned into the mammalian expression vector pCAGGS. Similarly, pCAGGS expression vectors encoding S1 or its subdomain S1_B_ of SARS (S1, residues 1 to 676; S1_B_, residues 325 to 533) and SARS2 (S1, residues 1 to 682; S1_B_, residues 333 to 527) C-terminally tagged with the Fc domain of human or mouse IgG or Strep-tag were generated as described before (*51*). Recombinant proteins and antibody 47D11 were expressed transiently in FreeStyle 293-F Cells (Thermo Fisher Scientific) and affinity purified from the culture supernatant by protein A Sepharose beads (GE Healthcare) or Strep-Tactin beads (IBA) purification. Purity and integrity of all purified recombinant proteins were checked by Coomassie-stained SDS–polyacrylamide gel electrophoresis.

### Enzyme-linked immunosorbent assay analysis of antibody binding to CoV spike antigens

Enzyme-linked immunosorbent assay (ELISA) was performed as described previously (*51*). Briefly, Nunc MaxiSorp plates (Thermo Fisher Scientific) coated with equimolar antigen amounts were blocked with 3% bovine serum albumin (Bio-Connect) in phosphate-buffered saline containing 0.1% Tween 20 at room temperature (RT) for 2 hours. Fourfold serial dilutions of mAbs starting at 10 μg/ml (diluted in blocking buffer) were added, and plates were incubated for 1 hour at RT. Plates were washed three times and incubated with horseradish peroxidase (HRP)–conjugated goat anti-human secondary antibody (ITK SouthernBiotech) diluted 1:2000 in blocking buffer for 1 hour at RT. An HRP-conjugated anti-StrepMAb (IBA) antibody was used to corroborate equimolar coating of the Strep-tagged spike antigens. HRP activity was measured at 450 nm using tetramethylbenzidine substrate (BioFX) using an ELISA plate reader (EL-808, BioTek). Half-maximum effective concentration (EC_50_) binding values were calculated by nonlinear regression analysis on the binding curves using GraphPad Prism (version 8).

### Pseudotyped virus neutralization assay

Neutralization with SARS2-S (GenBank: HD43416.1) and WIV16-S (GenBank: ALK02457.1) VSV pseudotyped viruses was performed as described previously (*51*). HEK-293T cells were transfected with pCAGGS expression vectors encoding SARS2-S carrying an 18–amino acid or WIV16-S carrying a 19–amino acid cytoplasmic tail truncation, respectively. One day after transfection, cells were infected with the VSV-G pseudotyped VSVΔG expressing the firefly (*Photinus pyralis*) luciferase. Twenty-four hours later, cell supernatants containing SARS2-S or WIV16-S pseudotyped VSV particles were harvested and titrated on African green monkey kidney VeroE6 (ATCC#CRL-1586) cells. In the virus neutralization assay, mAbs were threefold serially diluted and mixed with an equal volume of pseudotyped VSV particles and incubated for 1 hour at RT. The virus/antibody mix was subsequently added to confluent VeroE6 monolayers in a 96-well plate and incubated at 37°C. After 24 hours, cells were washed, and lysis buffer (Promega) was added. Luciferase activity was measured on a Berthold Centro LB 960 plate luminometer using d-luciferin as a substrate (Promega). The percentage of infectivity was calculated as the ratio of luciferase readout in the presence of mAbs normalized to luciferase readout in the absence of mAb. The IC_50_ value was determined using four-parameter logistic regression (GraphPad Prism version 8).

#### Flow cytometry–based antibody binding assay

Antibody binding to full-length SARS2-S epitope mutants on the cell surface was measured by flow cytometry. HEK-293T cells were seeded at a density of 2.5 × 10^5^ cells/ml in a T25 flask. After reaching 80% confluency, cells were transfected with an expression plasmid encoding full-length SARS2-S mutants with a C-terminal Flag tag, using Lipofectamine 2000 (Invitrogen). Twenty-four hours after transfection, cells were dissociated by cell dissociation solution (Sigma-Aldrich, Merck KGaA; catalog no. C5914). To detect total spike expression, cells were permeabilized by 0.2% saponin and subjected to anti-Flag tag antibody staining. For cell surface antibody binding measurement, intact (nonpermeabilized) cells were incubated with 20 μg/ml of 47D11, ACE2-Fc, CR3022 (target SARS2 RBD core), 49F1 (target SARS2-S1 outside RBD), and anti-Flag (Sigma-Aldrich, F1804) for 1 hour on ice, followed by incubation with 1:200 diluted Alexa Fluor 488–conjugated goat anti-human IgG antibodies (Invitrogen, Thermo Fisher Scientific, #A-11013) or goat anti-mouse IgG antibodies (Invitrogen, Thermo Fisher Scientific, #A28175) for 45 min at RT. Cells were subjected to flow cytometric analysis with a CytoFLEX flow cytometer (Beckman Coulter). The results were analyzed with FlowJo (version 10). FSC/SSC gates were used to select mononuclear cells. Control antibody staining was used to define positive/negative cell populations.

### Preparation of Fab-47D11 from IgG

47D11 Fab was digested from IgG with papain using a Pierce Fab Preparation Kit (Thermo Fisher Scientific), following the manufacturer’s standard protocol.

### Cryo-EM sample preparation and data collection

Three microliters of SARS2-S or SARS-S at 1.6 mg/ml was mixed with 0.85 μl of Fab 47D11 at 4 mg/ml and incubated for 50 s at RT. The sample was applied onto a freshly glow-discharged R1.2/1.3 Quantifoil grid in a Vitrobot Mark IV (Thermo Fisher Scientific) chamber preequilibrated at 4°C and 100% humidity. The grid was immediately blotted at force 0 for 5 s and plunged into liquid ethane. Data were acquired on a 200-kV Talos Arctica (Thermo Fisher Scientific) equipped with a Gatan K2 Summit direct detector and Gatan Quantum energy filter operated in zero-loss mode with a 20-eV slit width. To account for the preferred orientation exhibited by the spike ectodomains, automated data collection at tilts 0°, 20°, and 30° was carried out using EPU 2 software (Thermo Fisher Scientific) and data at tilt 40° using SerialEM (*66*). A nominal magnification of ×130,000, corresponding to an effective pixel size of 1.08 Å, was used. Movies were acquired in counting mode with a total dose of 40 e/Å^2^ distributed over 50 frames. A total of 4231 movies were acquired for SARS2 and 3247 movies for SARS-S, with defocus ranging between 0.5 and 3 μm.

### Cryo-EM data processing

Single-particle analysis was performed in Relion version 3.1 (*67*). The data were processed in four separate batches, corresponding to the stage tilt angle used for the acquisition. Drift and gain corrections were performed with MotionCor2 (*68*), Contrast transfer function (CTF) parameters were estimated using CTFFind4 (*69*), and particles were picked using the Laplacian picker in Relion (*67*). One round of two-dimensional (2D) classification was performed on each batch of data, and particles belonging to well-defined classes were retained. Subsequently, 3D classification was performed, using a 50-Å low-pass–filtered, partially open conformation as an initial model [EMD-21457; (*13*)], without imposing symmetry. All particles belonging to the Fab-bound class were then selected for 3D auto-refinement. Before merging the different batches, iterative rounds of per-particle CTF refinement, 3D auto-refinement, and postprocessing were used to account for the stage tilt used during data collection. The refined particle star files from each batch were then combined and subjected to a final round of 3D auto-refinement, per-particle defocus estimation, 3D auto-refinement, and postprocessing, both with and without imposed C3 symmetry. Overviews of the single-particle image processing pipelines are shown in figs. S7 and S8.

### Model building and refinement

UCSF Chimera (version 1.12.0) and Coot (version 1.0) were used for model building and analysis (*70*, *71*). The SARS2-S model, in the partially open conformation [one RBD up, Protein Data Bank (PDB) 6VYB] (*13*), was used for the spike and fitted into our density using the UCSF Chimera “Fit in map” tool (*70*). For SARS, a closed protomer of the PDB 6NB6 was used as a starting model (*72*). To build a model for the Fab, the sequence of the variable regions of the heavy chain (HC) and the light chain (LC) was separately blasted against the PDB. For the HC variable region, the corresponding region of the PDB 6IEB (human mAb R15 against RVFV Gn) was used (*73*). The LC variable region was modeled using the PDB 6FG1 as template (Fab Natalizumab) (*74*). For both chains, the query sequence of 47D11 was aligned to the template sequence. Sequence identity was particularly high (87 and 97% for the HC and LC, respectively). Phenix sculptor was used to create an initial model for the Fab chains (*75*), removing the nonaligning regions (notably the CDRH3). This model was fitted into our density, and the missing regions were built manually in the density map using Coot (*71*). Models were refined against the respective EM density maps using Phenix Real Space Refinement and Isolde (*76*, *77*) and validated with MolProbity (*78*) and Privateer (glycans) (*79*, *80*).

### Analysis and visualization

PDBePISA was used to identify spike residues interacting with 47D11 (*81*). Surface coloring of the SARS-CoV-2 RBD using the Kyte-Doolittle hydrophobicity scale was performed in UCSF Chimera (*70*). Volume measurements were performed using CASTp 3.0, using a probe radius of 1.2 Å (*82*). To color the 47D11-bound RBD surface according to each residue’s mean mutational effect on expression, the PDB file was populated with the mean mutation effect on expression values described by Starr *et al.* (*58*). The UCSF Chimera “MatchMaker” tool was used to obtain RMSD values, using default settings. Figures were generated using UCSF Chimera (*70*) and UCSF ChimeraX (*83*).

## SUPPLEMENTARY MATERIALS

Supplementary material for this article is available at http://advances.sciencemag.org/cgi/content/full/sciadv.abf5632/DC1

## Appendix

View/request a protocol for this paper from *Bio-protocol*.
